# Estimated dietary intake of essential elements from four selected staple foods in Najran City, Saudi Arabia

**DOI:** 10.1186/s13065-019-0588-5

**Published:** 2019-05-27

**Authors:** Hatem Mohamed, Parvez I. Haris, Eid I. Brima

**Affiliations:** 1 Faculty of Health and Life Science, De Montfort University, Leicester, LE1 9BH UK; 2 Department of Chemistry, College of Science, King Khalid University, Abha, 61413 Saudi Arabia

**Keywords:** EDI, ICP-MS, Essential elements, Rice, Wheat, Meat, Chicken, Najran, Saudi Arabia

## Abstract

The estimated dietary intake **(**EDI) of essential elements selenium (Se), zinc (Zn), manganese (Mn) and copper (Cu) has not been previously investigated for Najran city, Saudi Arabia. This type of information can be valuable for protecting public health. The aim of this study was to estimate the EDI of these elements. A food frequency questionnaire (FFQ) was completed by the study participants (n = 80) to obtain dietary intake of selected staple foods (rice, wheat, meat and chicken). The concentrations of Se, Zn, Mn and Cu in these staple foods were determined using inductively coupled plasma-mass spectrometry (ICP-MS). The ranges of concentrations (mg/kg, wet weight) were as follows: Se (0.07–0.24), Zn (3.91–20.89), Mn (0.63–14.69) and Cu (0.69–2.41). The calculated ranges of EDIs (mg/kg bw/day) for the essential elements were as follows: Se 9.55 × 10^−5^–5.75 × 10^−4^, Zn 1.33 × 10^−2^–5.83 × 10^−2^, Mn 1.49 × 10^−3^–3.31 × 10^−2^, Cu 1.65 × 10^−3^–5.42 × 10^−3^. The highest EDI for Cu and Mn came from wheat. In the case of Se and Zn, the foods that contributed the highest EDI were chicken and meat, respectively. The lowest EDIs were found for Se in wheat, Zn in rice and both Mn and Cu in chicken. The percentages (%) of provisional maximum tolerable daily intake (PMTDI) for Se, Zn, Mn and Cu were 13%, 11%, 14% and 3.4%, respectively when contributions from all the four classes of foods were combined. The percentage of the recommended daily allowance (RDA) derived from these foods were 80%, 20%, 17% and 5.6% for Se, Zn, Mn and Cu were, respectively. This raises the possibility of Cu deficiency in the Najran population. However, a total diet study and human biomonitoring study is needed in the future to fully assess if people in Najran city are at risk of deficiency or excessive exposure to trace elements.

## Introduction

Nutrition is an essential factor in health and disease [1]. The patterns of nutritional disorders in the developing world are further complicated by sociological changes, which can take place due to urbanisation and changing lifestyles [2]. Foods contain both essential and toxic elements that have significant effects on human health [3]. The human body needs different types of trace elements to perform nutritional, metabolic, and hormonal functions. The level of exposure to different elements depends on their intake, concentrations and distribution in foods [4].

Essential elements have beneficial health effects on human body and help in maintaining normal and complex physiological functions of the human body [5]. Essential elements such as Se, Zn, Mn, Cu and other major elements play important roles in human biology, physiology, metabolism and enzyme processes [5, 6]. Small quantities of essential elements are vital for human body’s growth and development, and they can be obtained through a balanced diet [5, 6].

An excessive intake or deficiency of essential elements may lead to health problems in human [5–7]. For instance, an excessive intake of Se leads to neurological disorders and generalised weakness [8], whereas deficiency in Se is associated with Kashin-Beck disease, cardiovascular diseases, hypothyroidism and weakened immune system [9–11]. It has been reported that excessive intake of Zn can lower the high-density lipoprotein (HDL) levels among healthy males [12]. Zn deficiency in humans is linked to loss of appetite, infant growth retardation, skin changes and immunological abnormalities [13]. In the case of Mn, excessive intake can cause health problems such as growth retardation, nausea and muscle weakness [14], whereas manganese deficiency is associated with changes in hair colour and loss of weight [15]. Similarly, long-term exposure to elevated levels of copper can lead to various conditions including Wilson’s disease and acute gastrointestinal problems [16]. Copper deficiency causes a range of disorders including anaemia, lymphoma, myelodysplastic syndrome and neutropenia [17].

Rice is a staple food for many populations in the world [18]. Rice is rich in micronutrients such as oryzanols, tocopherols, tocotrienols, phytosterols and dietary fibres like beta-glucan, pectin and gum [19]. Rice is grown in more than 100 countries, and there are different types of rice accounting for about 25% of the world’s food grain production including in continents such as Asia, Africa and America [20]. The elemental composition of the rice grain is potentially a critical factor that has an impact on human health. High consumption of white rice is associated with a significantly increased risk of Type 2 diabetes, mainly among Asian populations [21]. In Saudi Arabia, rice is considered as the dominant food source, and the per capita consumption is estimated at 42 kg/year [22].

Wheat grain is regarded as the second primary human food, after rice [23]. This cereal is composed of several different tissues, the germ, the endosperm, the thick cell-walled aleurone layer and the pericarp [23]. Wheat grains are also a major source of almost all vitamins including B-group vitamins, thiamine, riboflavin, niacin, pantothenic acid, pyridoxine, biotin, vitamin E and the carotenoids [24]. However, wheat consumption can also cause allergy in humans [25]. In Saudi Arabia, wheat consumption has increased by 2% in the period between the year 2011 and 2013 [26].

Meat is one of the essential food sources to humans and supplies the human body with proteins, amino acids and other essential minerals, required for tissue formation, growth and repair [27]. However, high intake of red meat has been linked to different diseases including cardiovascular disease, cancer and diabetes [28].

Chicken is one of the main staple foods in the world and represents a rich source of essential elements [29]. However, poultry meat can also be a source of exposure to pathogenic microbes and heavy metals [29, 30]. The consumption of beef and lamb in Saudi Arabia has decreased in the period 1985–2010 [26]. In contrast, the consumption of poultry has increased, and the Saudis consume 41.6 kg per capita of chicken annually [31].

From a public health perspective, it is crucial to assess the Saudis exposure to essential and toxic elements as they may play a role in the development of chronic diseases, which are on the rise in the Kingdom [32]. In Saudi Arabia in general, and in Najran city in particular, there is a lack of studies investigating the content of essential elements in the main staple foods and most of the previous studies had focused on calories and carbohydrate intake [33–35]. However, some recent studies are beginning to address this issue including studies on selected food from Saudi market and evaluation of trace metals in commonly consumed food in Saudi Arabia [36, 37].

This is the first study to investigate the concentration of essential elements in rice, wheat, red meat and chicken that are sold in markets in Najran city (Saudi Arabia) that are consumed by the local population. This study aimed to determine the estimated dietary intakes (EDIs) for the essential elements Se, Zn, Mn, and Cu in these main staple foods of the people in Najran city. The findings will be valuables for providing nutritional facts and information about the essential elements in these foods to improve the health of the Najran population and other regions of Saudi Arabia.

## Materials and methods

All aspects of the current study including location, study population, food consumption and average daily intake, collection of food samples, pre-treatment of food samples, digestion of food samples, elemental analysis and analytical method employed in the current study were same as reported in the recently published study for EDI of toxic elements [38]. Briefly, the study location was in Najran city in the south of Saudi Arabia, where 25 food samples of rice, wheat, loin of cooked meat (cow, sheep, goat and camel) and cooked breast of chicken were collected from a local market (El-Faisaliya market of Najran city). Food samples were analysed by using Inductively Coupled Plasma-Mass Spectrometry (ICP-MS) in Abha city at King Khalid University. For this study, ICP-MS was used for the analysis of essential trace elements in foods because it has a lower detection limit and high efficiency compared to techniques such inductively coupled plasma optical emission spectrometry (ICP-OES) or atomic absorption spectroscopy (AAS).

Additionally, a food frequency questionnaire (FFQ), the questionnaire comprises of simple questions to avoid the occurrence of misinterpreted questions. This step was followed by conducting a pilot test of the questionnaire by asking a group of 20 students and academic of Najran University to complete the questionnaire. The questionnaire was used to collect information from the 80 participants, a written consent was obtained from the participants in the study. The first section of the FFQ was used to collect information on socio-demographical variables including gender, age, marital status, occupation and educational status. The demographic parameters covered include gender, age group, marital status and educational level. The majority of the study population (55%) were females. The range of age is between 18 and 39 years for 50% of all the participants. Most of the studied population (57.5%) had a monthly income of SAR < 4000; more information is presented in Table 1.

**Table 1:** Demographic details of 80 participants from Najran city including gender age and other parameters

Demographic data (N = 80)	Frequency	Percent (%)
Gender		
Male	36	45.00
Female	44	55.00
Age (years)		
(18–39)	40	50.00
(40–59)	30	37.50
(60–79)	10	12.50
Marital status		
Single	26	32.50
Married	44	55.00
Divorced	3	3.80
Widowed	7	8.80
Education		
Illiterate	10	12.50
School level	25	31.30
University	43	53.80
Post-University	2	2.50
Occupation		
Government employee	24	30.00
Private sector employee	17	21.30
Student	9	11.30
Housewife	10	12.50
Unemployed	19	23.80
Other	1	1.30
Monthly income		
SAR < 4000	46	57.50
SAR 4000–7000	14	17.50
SAR 7000–10,000	15	18.80
SAR 10,000+	5	6.30

The FFQ included questions about portion sizes (in grams) of the different foods and consumption frequencies are presented in Table 2. Ethical approvals for the study were obtained from both De Montfort University (HLSFREC Ref: 1180) and Najran University ethics committee (23rd May 2013) before the beginning of the study.

**Table 2:** Intake of different foods in Saudi Arabia estimated from the FFQ

No.	Food type	Najran city (this study)	Saudi Arabia (other studies)
1	Rice	243 g	160 g—Riyadh City—Al-Kanhal et al. (1999) [65]
2	Wheat	161 g	298 g—Riyadh City—Nigatu et al. (2015) [66]
3	Meat	199.8 g	75 g—All thirteen administrative regions of Saudi Arabia—Moradi-Lakeh et al. (2017) [67] 116.69 g—Riyadh City—Al-Othman et al. (2012) [60] 47.60 g—Jeddah City—Al-Ahmary (2009) [57]
4	Chicken	170.4 g	75 g—All thirteen administrative regions of Saudi Arabia—Moradi-Lakeh et al. (2017) [67]
5	Vegetables	195 g	105 g—All thirteen administrative regions of Saudi Arabia—Moradi-Lakeh et al. (2017) [67] 203 g—Riyadh City—Al-Othman et al. (2012) [60]
6	Fruits	149.5 g	103 g—All thirteen administrative regions of Saudi Arabia—Moradi-Lakeh et al. (2017) [67] 90.54 g—Riyadh City—Al-Othman et al. (2012) [60] 289.50 g—Jeddah City—Al-Ahmary (2009) [57]
7	Fish	28.1 g	75 g—All thirteen administrative regions of Saudi Arabia—Moradi-Lakeh et al. (2017) [67]
8	Date	173 g	95.34 g—All regions of Saudi Arabia—Al-Shreed et al. (2012) [66] 100 g—All regions of Saudi Arabia—Kamel et al. (2007) [69]

### Analytical method

The analysis was carried out identically to that reported in a recent study [38]. Multi-element calibration standards were used and included the four elements (Se, Zn, Mn and Cu) at concentrations of 0.0, 1.0, 5.0, 10.0, 20.0 and 40.0 μg/L. Furthermore, an internal standard (scandium) was introduced online with a concentration of 100 μg/L. For quality control, a mixed standard (20 μg/L) of the measured elements was measured in each experiment after every five samples within the batch. The data processing was undertaken using Qtegra software (Thermo Fisher Scientific, Waltham, MA, USA).

### Quality control and quality assurance

The quality control for this study was performed by measuring known mixed concentrations (20 µg/L) of Se, Zn, Mn and Cu after every five samples. A mixed concentration of each element (20 µg/L) was spiked into randomly selected rice samples, and the recoveries for all measured elements were reported. For quality assurance, a certified reference material, NIES Certified Reference Material, Rice Flour-Unpolished, Low Level (Cd) (No10a NIES), was also measured.

### Health risk assessment for humans and estimated dietary intake calculation

Concentrations and EDIs were calculated for each element in the four staple foods based on the method described in a recent publication [38]. Briefly, the daily intake of each measured element in food was calculated based on the following equation:

1 $${\text{DI}}\left( {\upmu{\text{g}}} \right) = {\text{D}}\left( {\text{g}} \right) \times {\text{Cc}}\left( {\upmu{\text{g/g}}} \right)$$

where DI is the daily intake; D(g) = D is the average daily intake in all the food types (in g/day) and Cc is the calculated concentration (µg/g).

EDIs were expressed in mg/kg bw day was calculated as in the following equation:

2 $${\text{EDI}} = {{\left( {{{{\text{DI}}\left( {\upmu{\text{g}}} \right)} \mathord{\left/ {\vphantom {{{\text{DI}}\left( {\upmu{\text{g}}} \right)} {{\text{kg}}\;{\text{bw}}}}} \right. \kern-0pt} {{\text{kg}}\;{\text{bw}}}}} \right)} \mathord{\left/ {\vphantom {{\left( {{{{\text{DI}}\left( {\upmu{\text{g}}} \right)} \mathord{\left/ {\vphantom {{{\text{DI}}\left( {\upmu{\text{g}}} \right)} {{\text{kg}}\;{\text{bw}}}}} \right. \kern-0pt} {{\text{kg}}\;{\text{bw}}}}} \right)} {1000}}} \right. \kern-0pt} {1000}} = {\text{mg/kg}}\;{\text{bw}}\;{\text{day}}$$

where bw is the average body weight (in kg), average body weight in this study is 71.53 kg based on the data obtained from the participating volunteers. The calculation of the EDI is the same as the provisional maximum tolerable daily intake (PMTDI). The PMTDI value represents permissible human exposure of substance in food and drinking water [39].

Hazard quotients (HQs) for Se, Zn, Mn, and Cu in all 25 samples were calculated based on the following equation:

3 $${\text{HQ}} = {\text{EDIcalc/PMTDI}} .$$

In Eq. (3), EDIcalc is the EDI found in this study, and PMTDI is the provisional maximum tolerable daily intake (PMTDI) established by the WHO [40–43]. The PMTDIs are presented in Table 3.

**Table 3:** Provisional Tolerable Weekly Intake (PTWI) of Essential Elements (Se, Zn, Mn and Cu) and their provisional maximum tolerable daily intake (PMTDI)

No.	Elements	PTWI	PMTDI	References
1	Selenium (Se)	66 μg/kg bw/week	9.4 μg/kg bw/day	JECFA; WHO, 72 (2011) [70]
2	Zinc (Zn)	7 mg/kg bw/week	1 mg/kg bw/day^a^	JECFA; WHO, 26 (1982) [71]
3	Manganese (Mn)	2.5 mg/kg bw/week	0.36 mg/kg bw/day	JECFA; WHO, 26 (1982) [72]
4	Copper (Cu)	3.5 mg/kg bw/week	0.5 mg/kg bw/day^a^	JECFA; WHO, 26 (1982) [73]

^a^Calculated value based on PTWDI

## Results

### Quality control and quality assurance results

The recoveries for all measured elements in mixed quality control standard, which was measured after every five samples and was measured five times per batch (n = 15). The recoveries were as follows: Se (95.93%), Zn (97.60%), Mn (95.55%) and Cu (119.43%). Spiked recoveries were as follows: Se (99.86%), Zn (89.08%), Mn (99.09%) and Cu (100.96%). The result of the CRM (No.10a NIES) were as follows: Se (certified value 0.06, measured value 0.10 ± 0.01 μg/g), Zn (certified value 25.2 ± 0.8 μg/g, measured value 24.00 ± 0.03 μg/g), Mn (certified value 34.7 ± 1.8 μg/g, measured value 23.39 ± 0.08 μg/g) and Cu (certified value 3.5 ± 0.3 μg/g, measured value 3.37 ± 0.04 μg/g).

### Concentration of Se, Zn, Mn, and Cu in the four selected staple foods

The concentrations of the four essential elements (Se, Zn, Mn and Cu) were determined in the four selected staple foods samples. There is a substantial difference in the concentration of the four food minerals amongst the four selected four staple foods. Meat was found to have a high concentration of Zn (20.89 mg/kg). The highest Mn (14.69 mg/kg) concentration was found in wheat. The concentrations of Se (0.04 mg/kg) and Cu (0.69 mg/kg) were observed to be relatively low in wheat and chicken, respectively. The mean concentrations of Se, Zn, Mn, and Cu (mg/kg) in the 25 samples of rice, wheat, red meat and chicken are presented in Table 4.

**Table 4:** Mean concentrations (µg/g) mean ± SD of elements (Se, Zn, Mn and Cu) measured in 25 samples of the four food types (rice, wheat, meat and chicken) in dry weight samples: rice (n = 8), wheat (n = 5), meat (n = 4) and chicken (n = 8)

No.	Food type	${\text{Average}}{:}\;\left( {\frac{Dry weight}{Wet weight}} \right)$	Essential elements (µg/g)			
			Se	Zn	Mn	Cu
1	Rice	0.92	0.13 ± 0.09	9.26 ± 4.20	10.07 ± 11.87	0.8 ± 0.32
2	Wheat	0.88	0.19 ± 0.11	18.99 ± 6.97	24.53 ± 9.89	1.47 ± 0.65
3	Meat	0.26	0.06 ± 0.01	11.58 ± 1.58	0.17 ± 0.07	0.16 ± 0.10
4	Chicken	0.39	0.62 ± 0.14	18.42 ± 3.19	1.60 ± 1.13	1.77 ± 0.38

Average of dry weight/wet weight was also reported

### Daily intake (DI) of essential elements

The average DI (µg/day) of Se, Zn, Mn, and Cu estimated from the four food categories (rice, wheat, meat and chicken) for an adult Najran person are reported in Table 5. The mean average DI (µg/day) was 23.96, 2401.38, 887.05 and 282.25 for Se, Zn, Mn and Cu, respectively. In the case of Zn, meat provides the highest intake of this element out of the four selected staple foods in this study (4173.55 µg/day). Furthermore, the daily intake of Se was the lowest from wheat (6.83 µg/day), whereas Mn and Cu had the highest daily intake from wheat amongst the four staple foods. Data presented in Table 5 also show that the overall highest DI of Zn comes from meat, followed by wheat.

**Table 5:** Dietary intake (DI) of the essential element (Se, Zn, Mn and Cu) in 25 samples of the four food types (rice, wheat, meat and chicken)

DI of the essential elements (µg/day)				
Food type	Se	Zn	Mn	Cu
Rice	37.5	2502.5	2667.5	215.0
Wheat	25.5	1364.3	2118.8	121.5
Meat	48.0	9456.0	132.0	108.0
Chicken	138.6	4274.6	437.8	413.6
Water^a^	2.83	94.10	0.18	1.73
Total	252.43	17.691.5	5356.28	859.83

^a^Data taken from Brima study 2017

### Estimated provisional maximum tolerable daily intakes (PMTDI)

**Fig. 1:**
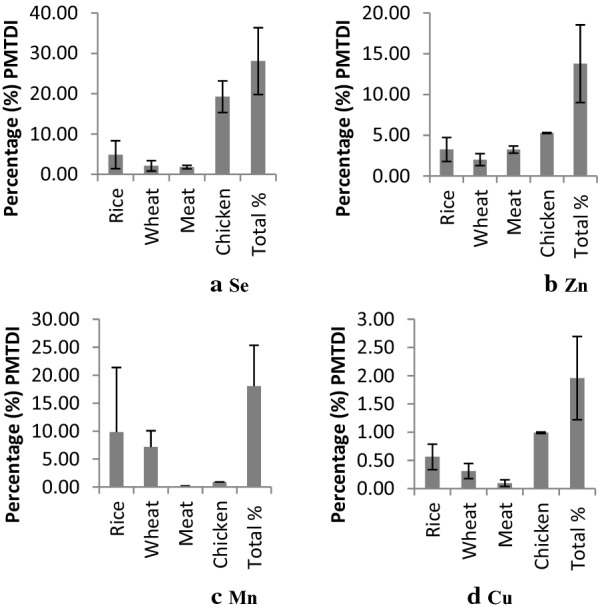
Percentage (%) PMTDI contributions from each food class and all food classes combined—for each element; **a** (Se), **b** (Zn), **c** (Mn) and **d** (Cu)

Figure 1 presents the percentages (%) of the estimated provisional maximum tolerable daily intakes (PMTDI) for Se, Zn, Mn and Cu. The percentages (%) PMTDI explain the contributions from each food class and all food classes combined for each element. For each of the elements, the % PMTDI is above 11%, except for Cu. The lowest percentage of the PMTDI is seen for Cu with 3.4% when contributions from all the four classes of foods are combined.

### Estimated dietary intake (EDI) and recommended daily allowance (RDA) of essential elements

Table 6 illustrates the estimated dietary intake (EDI) of essential elements and the hazard quotient (HQ) for Se, Zn, Mn and Cu. The decreasing order of EDIs (mg/kg bw/day) was as follows: Zn (5.83 × 10^−2^) in meat, Mn (3.31 × 10^−2^) in wheat, Cu (5.42 × 10^−3^) in wheat and Se (5.75 × 10^−4^) in chicken. The HQ for each of the essential elements does not exceed one.

**Table 6:** Estimated dietary intake (EDI) of the essential element (Se, Zn, Mn and Cu) in 25 samples of the four food types (rice, wheat, meat and chicken)

EDI of the essential elements (µg/kg bw/day)				
Food type	Se	Zn	Mn	Cu
Rice	0.46	32.56	35.41	2.81
Wheat	0.20	20.03	25.88	1.55
Meat	0.17	32.38	0.48	0.48
Chicken	1.81	52.81	3.14	4.95
Total	2.64	137.78	64.91	9.79

**Fig. 2:**
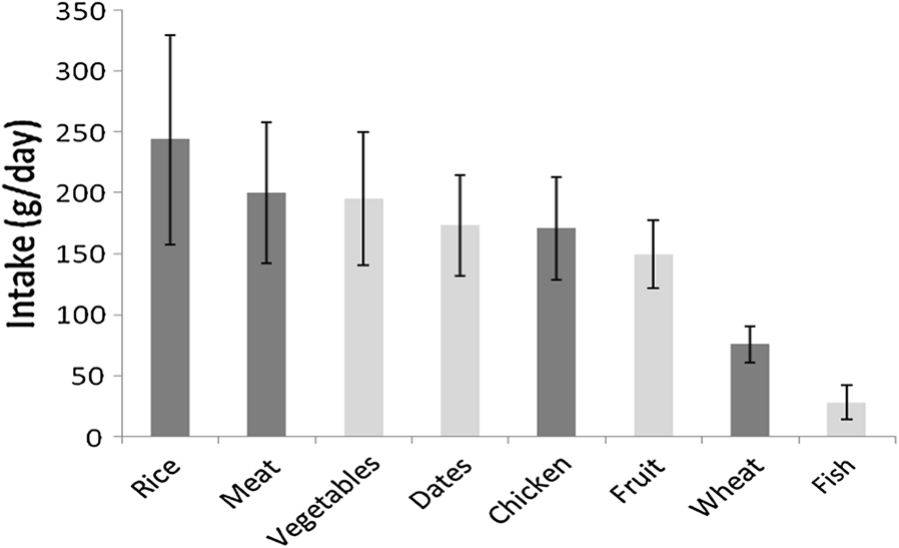
Average daily intakes of foods by food group in the Najranian population total diet (dark grey) food groups investigated in this study; (light grey), food groups not investigated in this study. The values are means, with standard deviations represented by vertical bars

Figure 2 presents the four measured essential elements (Se, Zn, Mn and Cu) in the four common staple foods regarding average daily intake. The daily intake of each of the four elements for each of the four foods was calculated. As can be seen, the four foods analysed supplies more than 2401.38 µg/day of Zn. These findings come as a result of a higher daily dietary intake from chicken, followed by meat. The provisional maximum tolerable daily intake (PMTDI %) for the measured elements was calculated. Mn has the highest PMTDI (14%) followed by Se (13%). Copper has the lowest PMTDI that contributed to 3.4% of the total PMTDI. Taking into considerations the daily intakes measured for the investigated elements, the percentage of the RDAs, derived from the four foods (rice, wheat, meat and chicken), for Se, Zn, Mn and Cu were 80%, 20%, 17% and 5.6%, respectively.

## Discussion

This study was carried out to determine the EDI of essential elements (Se, Zn, Mn and Cu) from four selected staple foods (rice, wheat, meat and chicken) of a population in Najran city, Saudi Arabia. This study provides valuable nutritional risk assessment based on EDIs of the essential elements in this population. There is a little information available regarding the EDIs of essential elements in the main four selected staple foods in Saudi Arabia including Najran city.

### Concentration of essential elements in four selected foods

The results of this study showed the concentrations of the elements in all food samples (rice, wheat, meat and chicken) as follows: Se (0.07–0.24 mg/kg), Zn (3.91–20.89 mg/kg), Mn (0.63–14.89 mg/kg) and Cu (0.69–2.41 mg/kg). The range of concentration of the elements in all the food samples in our study is within the range of the WHO recommended limits [44].

### Essential elements in rice

In this study, the mean level of concentrations (mg/kg) of all rice samples for Se, Zn, Mn and Cu were 0.07, 3.91, 4.32 and 1.44, respectively. Our results for rice samples were lower than the results, except for Se, from a study in Bangladesh that had measured the mean concentrations (mg/kg) levels of essential elements in rice Se (0.03), Zn (13.18), Mn (4.65) and Cu (1.99) [45]. Our results differ from the findings of a study from Sweden, which found concentrations (mg/kg) for Se (0.10), Zn (15.00), Mn (16.00) and Cu (1.90) [46]. We detected lower concentrations for all the measured elements compared to the latter study. Our findings are similar to the results of a study on rice samples from Qatar market, which had found the concentrations level for Se to be in the range 0.006–0.42 mg/kg [43]. Moreover, their Zn (2.79–29.90 mg/kg) concentration was similar to our rice samples. The variances noted in results between our study and other studies from the literature could be attributed to several factors such as the water used in irrigation as well as the type of soil where the rice was cultivated etc. [47].

### Essential elements in wheat

The findings of this study reveal that the mean concentrations (mg/kg) of Se (0.04), Zn (11.19), Mn (14.69) and Cu (2.41) in wheat samples were all lower than recommended WHO values [44]. A study in Bangladesh analysed 30 different kinds of wheat by ICP-MS reported the concentrations level of Se (1.894 mg/kg), Zn (0.245 mg/kg), Mn (0.0012 mg/kg) and Cu (0.209 mg/kg) [45]. The authors suggested that these levels posed no health risks to human [45]. Another study on wheat grains from Iran had reported lower concentrations of Cu (0.86 ± 0.19 mg/kg) and Zn (1.39 ± 0.5 mg/kg) than our values. Both reported values were below the WHO standard [44, 48]. Moreover, geographic differences between samples and nature of soil and water can cause variation among results of different studies [49]. Also, the concentrations of elements detected do not exceed the recommended values of the WHO [44].

### Essential elements in meat

For the meat samples, the mean concentrations (mg/kg) were as follows: Se (0.12), Zn (20.89), Mn (0.95) and Cu (1.71). A recent study from Australia on sheep meat found that the concentrations for Cu was 0.74 mg/kg [50]. Their results (mg/kg) for muscle, liver and kidneys were 0.014, 1.05 and 0.44 for Zn, respectively. The mean levels (mg/kg) for Se in muscle, was 0.09 [50]. Their results are similar to our results except for Zn which is lower than ours. Another study on essential elements in red meat, in the Canary Islands (Spain), found the mean concentrations of Cu (0.87 mg/kg), Zn (33.15 mg/kg) and Mn (0.11 mg/kg). With the exception of Zn, these are all lower than our results. The concentrations of the elements in these studies were not exceeding the international limits and posed no health risks [44, 51]. Our study for cooked meat varies from the results of a study in Brazil, on beef meat cooked in water that had found the concentrations (165 ± 2 mg/kg) for Zn and (1.2 ± 0.6 mg/kg) for Cu [52]. Compared to the latter study, the concentration of Zn was higher than our results, while the Cu levels were similar to our results. Despite the fact that our study and other studies discussed here were for cooked meat, one might argue that differences in the concentration of elements can be attributed to differences in the type of food used in feeding the animals as well as differences in age and environment [53].

### Essential elements in chicken

The results of this study showed that in all the studied chicken samples, the mean concentrations (mg/kg) of the essential elements of Se (0.24), Zn (7.21), Mn (0.63) and Cu (0.69), respectively. The findings of our study were within the ranges of the results of a study from Turkey on chicken samples that determined the concentration of Cu (0.10–114 mg/kg), Se (0.10–0.91 mg/kg) and Mn (0.05–3.91 mg/kg). They concluded that their results were within the recommended limit and posed no risk for human consumption [54]. In a recent study from Brazil, it was found that the concentrations of different elements in chicken samples were 0.65 ± 0.1 mg/kg and 29 ± 1 mg/kg for Cu and Zn, respectively [52]. Their results showed that Cu was similar to our results and Zn was higher than ours. These differences indicate that chicken samples originating from different locations can have widely different elemental contents, which could be due to nature of the feeding ingredients as well as the difference in age and environment [55].

### Daily intake (DI)

This study results showed that the daily intake (DI) of Se, Zn, Mn and Cu in the four food types (rice, wheat, meat and chicken) varies widely. The mean daily intake (µg/day) for essential elements in rice, wheat, meat and chicken were Se (6.83–41.12), Zn (949.02–4173.55), Mn (106.87–2365.15) and Cu (118.12–387.51), respectively (Table 5). There are difference and similarity between our study findings and other studies in the literature [56, 57]. These variations in the result could be due to the fact only four types of foods were investigated in our study, while other studies investigated many different types of food categories or carried out a total diet study. In the future more studies are needed to assess the total daily intake of essential elements in Najran by including other foods not included in our study.

### Estimated dietary intake (EDI) and recommended daily allowance (RDA)

The findings of this study showed the range of EDI (mg/kg bw/day) of the essential elements vary widely in the main four staple foods rice, wheat, meat and chicken. It is not possible to directly compare our EDI to those reported in the literature since our study was based on the intake from only four types of foods. The variations in the EDI between our study and other studies from Qatar, Korea, Saudi Arabia, Spain and Italy can be attributed to different factors [56, 58–61]. For example, the condition of the soil, water used for irrigation and content of minerals in animal feed is unlikely to be similar between different countries, and it affects the level of concentration of these elements on the main staple foods of these countries [62]. Other factors may be because of environmental factors such as for example growing rice in polluted soils [63]. Additionally, the quantities of main staple foods that are commonly consumed vary among different populations, which will also lead to differences in the EDI. Besides, other studies have included more food types (for example thirteen food types) [58], whereas in this study we only focused on four staple foods consumed in one city (Najran) in Saudi Arabia. Moreover, our study was focused on two food categories, cereal (rice and wheat) and meat (poultry and red meat).

We focused on determining if the four foods analysed in this study provides sufficient quantities of the essential elements to meet the daily requirements. The four staple foods in this study contribute to PMTDI with the following percentages related to the essential elements; Se (13%), Zn (11%), Mn (14%) and Cu (3.4%) as presented in Fig. 1. The PMTDI of these four elements shows no risk of exposure to excess of essential elements from the four foods investigated.

The percentage of the recommended daily allowance (RDA) for Se, Zn, Mn and Cu were 79.8%, 20.1%, 17.7% and 5.6%, respectively. Se represents almost about 80% of the RDA from the four foods. It is highly likely that the population in Najran should be able to receive the remaining 20% of the RDA from other foods that were not investigated in this study but are rich in Se including cruciferous vegetables, fruits and seafood [64]. The RDA estimated in our study for Zn was 20%, which means 80% of Zn will need to be derived from other foods to avoid deficiency of this important element among Najran population. This needs to be investigated in the future to check if there is a possibility of Zn deficiency. If Zn intake is low in the population, after a total diet or human biomonitoring study, the population will need to consume foods that are rich in this element such as milk and dairy products, legumes and seafood [65, 66].

Concerning Mn, the four investigated staple foods provided only 17% of the RDA. Thus, people will need to obtain the remaining amount of the total RDA from other foods that are rich in Mn. In addition to meat and poultry, Mn can be found in foods such as grains, tea, nuts and fish [66, 67]. However, it is known from our food frequency questionnaire in this study that the Najran people do not consume a high amount of fish. Therefore, people in Najran could obtain Zn and Mn by increasing their consumption of other foods such as fish and vegetables. Further, in our study, we estimated that only 5.4% of the RDA of Cu is derived from the four staple foods investigated. This value is very low compared to the international RDA set by the WHO [68]. The low intake of Cu from these foods may lead to a possibility of a deficiency of Cu in the Najran population. There are some disease that are prevalent in the population that may be linked to Cu deficiency such as anemia [69]. A study by Al Faran et al. [70] had reported that anemia is highly prevalent among adults pregnant and non-pregnant women in Najran area. Also, another study by Al-Muhaimeed et al. [71] had confirmed that the prevalence of sickle cell disease in Najran is higher than some other part of Saudi Arabia. This could be due to socio-economic, educational level and dietary reasons [74]. Cu deficiency can be avoided by consuming foods that are rich in Cu from other sources including, but not limited to, drinking water, tea, apple juice, milk, meat-poultry, eggs and cereals [75].

To obtain a full picture of the EDIs and RDAs, a comprehensive study is needed in the future by including the analysis of other foods that were not investigated in the current study (for example vegetables, fruits, fish and water). Such kind of study may help the public health professionals and policymakers to advise people on the healthy nutrition and how they can obtain an optimum intake of the essential elements.

## Conclusion

This is the first study to have determined the intake of essential elements (Cu, Zn, Mn and Se) from foods in a population from Najran by combining data from a food frequency questionnaire with analysis of foods using ICP-MS. The content of these four essential elements, in four commonly consumed foods (rice, wheat, meat and chicken), was determined. The EDIs for the measured essential elements in the foods analysed in this study did not exceed the recommended values set by WHO. The highest EDIs for measured essential elements from the four investigated foods were Cu and Mn from wheat, Se from Chicken and Zn from meat. However, the lowest EDIs input was Se from wheat, Zn from rice, and both Mn and Cu from Chicken. The four investigated foods altogether provided a relatively high percentage of RDA for Se, however, the % RDA for Mn, Cu and Zn were quite low. The results of this study are beneficial for the public health professionals and provide a basis for further examining the diet of the population in Najran in more detail in order to develop safety policies and to evaluate health risk related to dietary intake of essential elements. A total intake of elements from different foods in Najran as well as human biomonitoring studies will be considered in the future.

## Data Availability

The data that support the findings of this study are available from the corresponding author (E.B.) upon reasonable request.

## Abbreviations

BW: body weight
CC: calculated concentration
Cu: copper
DI: daily intake
EDI: estimated dietary intake
EDIs: estimated dietary intakes
FFQ: food frequency questionnaire
HQ: hazard quotient
HDL: high density lipoprotein
ICP-MS: inductively coupled plasma-mass spectrometry
ICP-OES: inductively coupled plasma optical emission spectrometry
Mn: manganese
PMTDI: provisional maximum tolerable daily intake
PTDI: provisional tolerable daily intakes
RDA: recommended daily allowance
SAR: Saudi Arabia Riyals
Se: selenium
WHO: World Health Organization
Zn: zinc
